# Binderless, All-Lignin Briquette from Black Liquor Waste: Isolation, Purification, and Characterization

**DOI:** 10.3390/molecules26030650

**Published:** 2021-01-27

**Authors:** Yati Mardiyati, Emia Yoseva Tarigan, Pandji Prawisudha, Silvia Mar’atus Shoimah, Raden Reza Rizkiansyah, Steven Steven

**Affiliations:** 1 Material Science and Engineering Research Group, Faculty of Mechanical and Aerospace Engineering, Institut Teknologi Bandung, Jl.Ganesha 10, Bandung 40132, Indonesia; yosevaemia@gmail.com (E.Y.T.); silvia.maratus999@gmail.com (S.M.S.); raden.reza.rizkiansyah@gmail.com (R.R.R.); steven@material.itb.ac.id (S.S.); 2 Energy Conversion Research Group, Faculty of Mechanical and Aerospace Engineering, Institut Teknologi Bandung, Jl.Ganesha 10, Bandung 40132, Indonesia; pandji@termo.pauir.itb.ac.id

**Keywords:** acid precipitation, binderless briquette, black liquor waste, lignin

## Abstract

Lignin isolated from black liquor waste was studied in this research to be utilized as binderless, all-lignin briquette, with a calorific value in the range of 5670–5876 kcal/kg. Isolation of lignin from black liquor was conducted using the acid precipitation method. Sulfuric acid, citric acid, and acetic acid were used to maintain the pH level, which varied from 5 to 2 for the precipitation process. The influence of these isolation conditions on the characteristic of lignin and the properties of the resulted briquette was evaluated through the Klasson method, proximate analysis, ultimate analysis, Fourier Transform Infrared (FTIR), adiabatic bomb calorimeter, density measurement, and Drop Shatter Index (DSI) testing. The finding showed that the lignin isolated using citric acid maintained to pH 3 resulted in briquette with 72% fixed carbon content, excellent 99.7% DSI, and a calorific value equivalent to coal-based briquette.

## 1. Introduction

Inevitable depletion of non-renewable and limited fossil fuel in the future, such as petroleum, coal, natural gas, etc., is an issue, since 80% of world’s energy generation was still dependent on the utilization of these fossil fuels [1,2,3]. The concern over this problem and advancing demand for energy with the increase of global human population and development of commercial and industrial activities, are raising awareness on the development of alternative energy source. Application of rapidly generated byproducts like municipal solid waste or renewable resources such as biomass are some alternatives that were researched as fuel for energy generation in the future [1]. Some biomass, for example, spruce sawdust [4], water hyacinth [5,6], coffee residue [7], eucalyptus leaves [7], and lignin [8] was studied to be developed into fuel in previous studies. Lignin is a naturally occurring polymer that exists in plants. Lignin could be obtained through extraction from plants or the more readily available black liquor.

Black liquor is a byproduct of the kraft papermaking process that yields as dry weight around 170 million tons annually [9]. Black liquor is harmful to the environment as it could contain compounds such as methyl mercaptan and dimethyl sulfide [10]. To overcome this problem, some measure was conducted to prevent the buildup of black liquor waste in an environmentally harmful manner. Utilization as fuel in a recovery boiler to generate electricity and steam in pulp mills was presently applied as energy conservation and one of the viable options to reduce the black liquor waste. Direct utilization of black liquor as fuel usually occurs in concentrated form, with a 65–70% solid content [11,12]. It has a typical energy value as a fuel of around 3225.5–3891 kcal/kg, which allows providing up to 12% of the energy required in the industry mentioned above [11,12].

Black liquor consists of organic and inorganic constituents. The organic constituent is the part that contributes to the generation of energy during its combustion. Existence of inorganic constituents that could reach as high as 40–50% become the main problem in the direct utilization of black liquor as fuels, as it is rendered into molten smelt and low melting point ash [11]. Accumulation of ash and smelt inside the boiler greatly reduces its thermal efficiency and also generates a corrosive environment that could affect the lifespan of metal-based equipment [11]. Isolation of its organic constituent, the actual fuel in black liquor, could be a viable pathway to greatly improve its application as fuel.

Lignin is the largest organic constituent within black liquor, which covers around 38–47% of its dry mass, depending on the type of wood [9,10]. Up to 80% of lignin in black liquor could be recovered [12]. Lignin molecules consist of a large number of carbon atoms, contributing to its outstanding potential as a biofuel. In addition to being a biofuel, lignin is also reported to have potential utilization in some other applications such as electrochemical sensors, biosensors, fillers, antioxidants, bacterial growth suppressants [13], wastewater adsorbents [14], packaging, tissue engineering, and abrasive tools [15]. The application of lignin in biofuel application is drawing attention, as it has a heat value of around 5975.1—6333.7 kcal/kg, almost two times that of the concentrated black liquor [11]. The high energy potential of lignin makes it a promising substance to be developed into green energy material. Lignin-based fuel could be considered as green, as it is obtained from renewable raw materials like trees and plants, and especially from black liquor waste in the paper and pulp industry.

Lignin can be isolated from black liquor by reducing the pH of the system. Sulfuric acid is regarded as effective for use in the lignin precipitation process, as it could bring the system pH to as low as 2 [16]. A lower pH is required to allow protonation on phenolic and carboxylic functional groups in lignin, and allow it to separate from black liquor [16]. However, sulfuric acid utilization has some drawbacks, such as the release of harmful gases that could lead to an environmental problem and which is carcinogenic to humans [17,18]. The utilization of a weaker acid is considered to be viable to overcome these problems.

Lignin is reported to be used as a binder in briquette application [1,2,19,20,21,22]. Previous research studies utilized lignin as a binder for anthracite-based briquette [23], organic municipal solid waste [24], and wood [4,25]. It was also reported that lignin can serve both as a binder and fuel, improving both the physical and heat properties of the briquette. Briquette is densified particles or loose fuel materials. Briquetting involves pressure to compact the particles or loose fuel materials into smaller volume agglomerates that are capable of remaining compressed [1]. It has some advantages over direct utilization of biomass as fuel, such as a higher heat content and a more compact size, which gives ease of storage space for use and transport [26]. Briquettes are usually produced using coal or biomass such as agricultural waste, with binder occasionally added to the system. Binder is needed in some cases where the material cannot form a strong densified form with a role to facilitate inter-particulate bonding, thus allowing the densified form to be formed [1,21]. The organic-based binder is preferred to the inorganic-based binder, even though the latter form briquette with a higher compressive strength, compaction ratio, and is more hydrophobic [1]. This is because the addition of inorganic substances tends to increase the ash content and burn out temperature, and reduces the calorific value of the resulted briquette [1,26]. Despite their flexibility, binder-based briquettes have some disadvantages such as non-uniform combustion properties and decrease in compacting properties at high temperatures, as the influence of different thermal behavior between the binder and fuel material [19,20].

Utilization of the binderless briquette, a type of briquette compacted without binder, is more advantageous in these cases, although material that could be used in this type of briquette is limited as it should be able to form a strong attraction between each particle. Lignin, which is usually used as the binder, should be capable of being compacted into an all-lignin, binderless briquette. This capability was possible since it has both significant properties as fuel and the ability to bind with each other through a hydrogen bond, which is contributed from the hydroxy group (-OH) in lignin.

In this research, lignin was isolated and purified from black liquor by using the acid precipitation method and was utilized as a binderless, all-lignin briquette. Isolation of lignin was conducted using citric acid and acetic acid, a more environment friendly alternative to the stronger sulfuric acid. Citric and acetic acid was reported to have the ability to precipitate lignin [17]. Effect of pH and type of acid on the purity of obtained lignin and the properties of briquette were evaluated. To the best of our knowledge, the utilization of lignin into a single component binderless briquette was not reported, which mostly applied as a binder. Evaluation of briquette properties was conducted through density measurement, proximate and ultimate analysis, calorific value test, and drop shatter test.

## 2. Results and Discussion

### 2.1. Effect of pH and Acid Type on the Lignin Isolation Process

Isolation of lignin from black liquor was conducted by the acid precipitation method. Sulfuric acid, citric acid, and acetic acid were utilized to reduce the pH level of black liquor and allow lignin to precipitate. The pH level of black liquor was reduced from basic to acid level, using the corresponding acids. The precipitation condition was maintained throughout all levels of pH from 5 until the lowest pH possible, with the addition of the acids.

**Figure 1:**
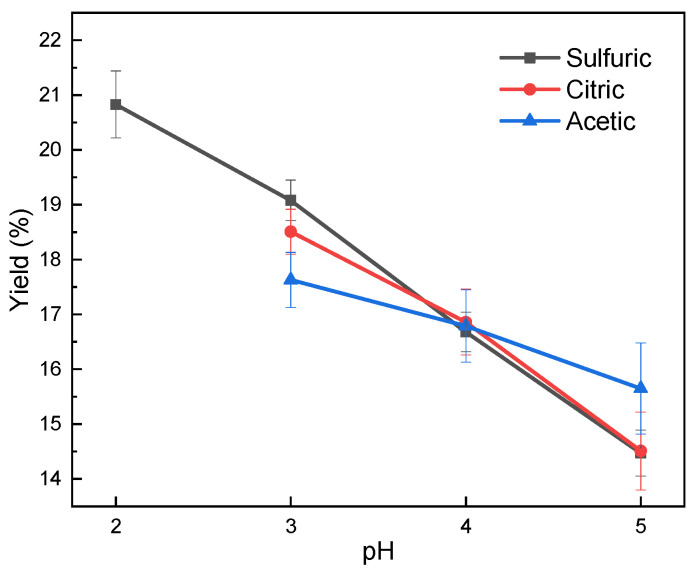
Yield of the isolated lignin in various pH and acid types.

Figure 1 shows the effect of various pH levels and acid type on the yield of the isolated lignin. The yield of the lignin samples that were precipitated using acetic acid, citric acid, and sulfuric acid increased with the decrease of pH level. The highest yield obtained was 20.83%, which was precipitated using sulfuric acid at pH 2. The yield was comparable to previous studies that could isolate lignin from black liquor, with a yield of around 16–23% [27]. The precipitation using acetic acid and citric acid with pH 2 could not be achieved, due to its limited acid strength. At pH 4, the three acids resulted in a similar yield percentage. This indicated that the pH level had a significant effect than the type of acid on the yield of precipitated lignin, which was in line with previous studies that stated that the yield of lignin increased with a decrease in the pH of the precipitation process [16]. The lower the pH level, the higher the H^+^ ion concentration in the solution. Increase of H^+^ ion inducing protonation of negatively-charged phenolic group of lignin and render its surface charge neutral [28]. Neutralization of lignin surface charge reduces the repulsive force between molecules and leads the intermolecular attraction to become dominant. Those conditions cause lignin molecules to aggregate and precipitate.

**Figure 2:**
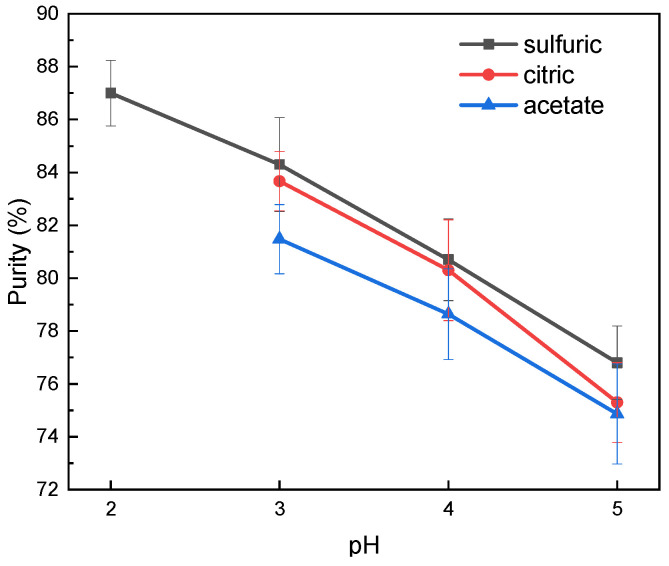
The purity of isolated lignin in various pH and acid types.

The isolation process through precipitation was followed by washing in boiling water to purify the isolated lignin from impurities. The purity of precipitated lignin was evaluated using the Klasson method. The purity increased with the decrease of pH during the precipitation process, as presented in Figure 2. The highest lignin purity was 87%, which resulted from isolation using sulfuric acid in pH level 2. Lignin impurities in the precipitate were caused by the presence of several substances in the black liquor that attached to the lignin, which could include inorganic and organic substances. The main inorganic substances that exist in black liquor are sodium sulfide, sodium carbonate, sodium sulfate, sodium hydroxide, sodium thiosulfate, and sodium chloride [29]. These substances were difficult to dissolve in water. Some possible organic substance impurities were extractives, acetic acid, formic acid, and methanol. These substances are contained in black liquor in relatively high amounts, which was around 25–35% for extractives; 5% for acetic acid; 3–5% for formic acid; and 3% for methanol [29]. Acid soluble lignin is not considered to be included as the final lignin content because its smaller molecular weight makes it more difficult to recover. The recovery of these fraction of lignin would increase the production cost, if applied on a larger scale.

### 2.2. Effect of Acid Type on the Proximate Value of Isolated Lignin

The result of the proximate analysis is presented in Table 1. Proximate analysis result consists of moisture content, volatile matter, ash content, and fixed carbon. In this study, we used SNI 4931–2010 as a standard reference to evaluate the quality of lignin briquette created in this research [30].

**Figure 3:**
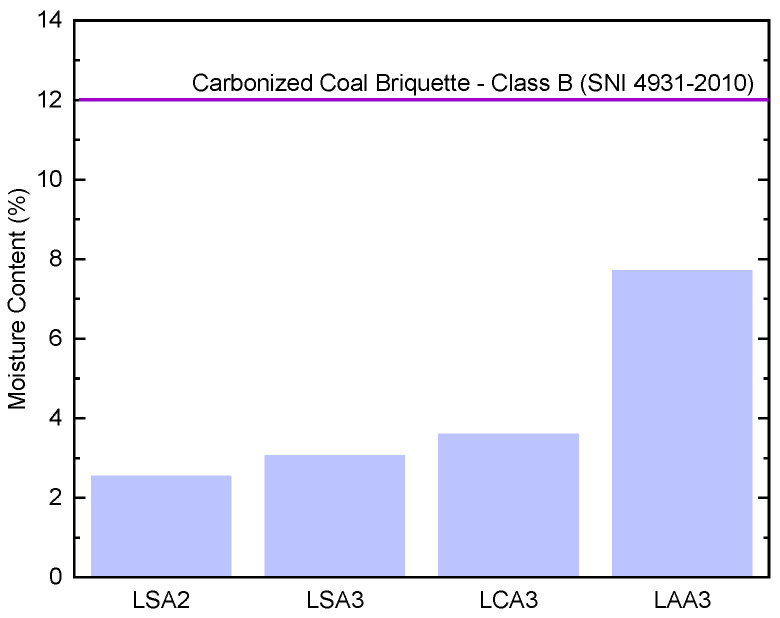
Moisture content of lignin LSA2, LSA3, LCA3, and LAA3 compared to carbonized coal briquette from the SNI 4931–2010 requirement.

The result showed that lignin isolated using sulfuric acid at pH 2 (LSA2) had the lowest moisture content. Evaluation of the moisture content of the samples, as presented in Figure 3, showed that all samples had a moisture content lower than that required in the SNI 4931-2010 standard.

**Figure 4:**
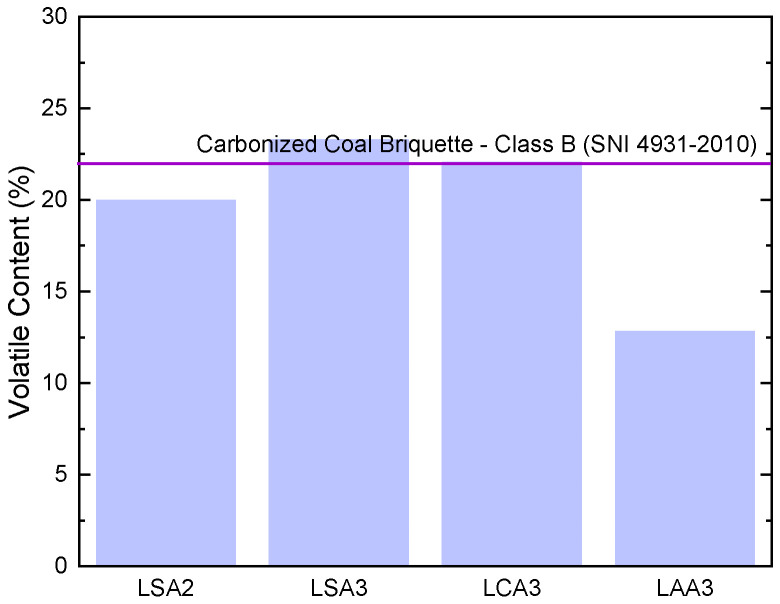
Volatile content of lignin LSA2, LSA3, LCA3, and LAA3 with carbonized coal briquette SNI (4931–2010).

**Figure 5:**
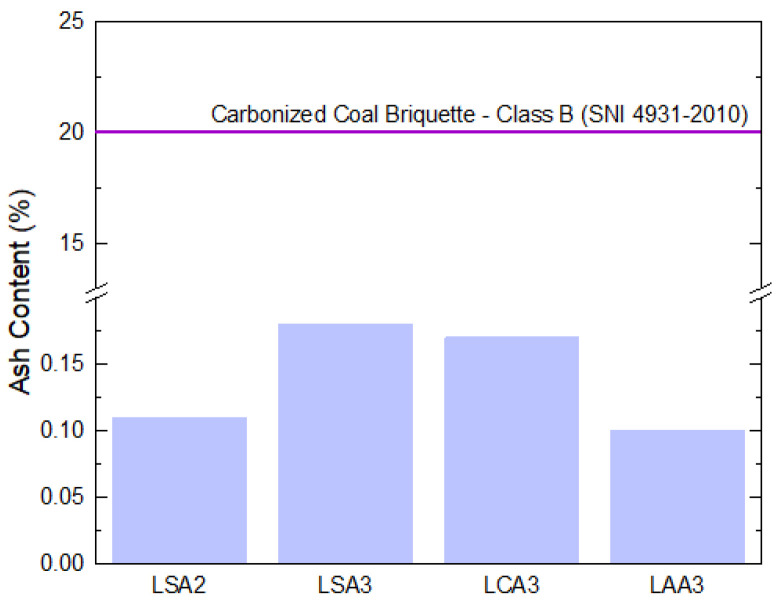
Ash content of the lignin LS2, LS3, LCA3, and LAA3 compared to the carbonized coal briquette SNI 4931–2010.

The lowest volatile content of the precipitated lignin resulted from isolation using acetic acid to pH 3 (LAA3), reached as low as 12.80%, as presented in Figure 4. The presence of volatile content was possibly caused by impurities remaining after the purification process. Results also showed that the volatile content of LSA3 and LCA3 were slightly above the allowed volatile content by the SNI 4931–2010 standard. Utilization of acetic acid at pH 3 for precipitation of lignin also resulted in the lowest ash content, which was only 0.10%. Overall evaluation of the ash content of all samples was in good agreement with the SNI 4931–2010 standard requirement, as presented in Figure 5.

**Figure 6:**
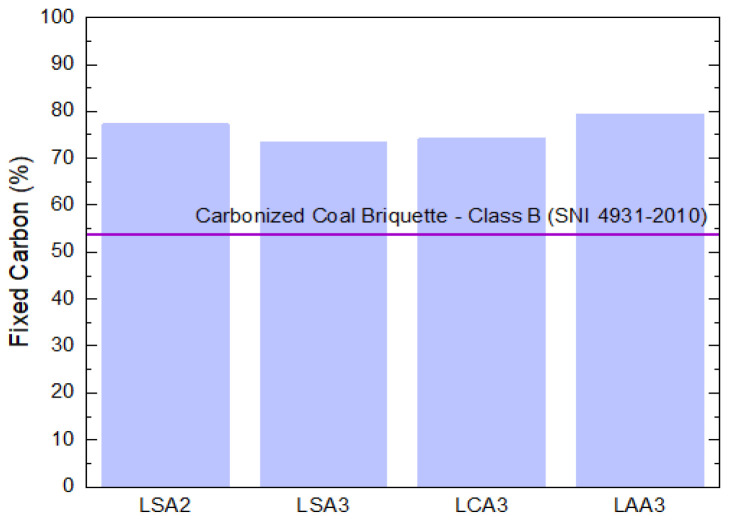
Fixed carbon of LS2, LS3, LAS3, and LAA3 samples compared to the carbonized coal briquette SNI 4931–2010.

The fixed carbon content of the precipitated lignin samples, as compared to the carbonized coal briquette of SNI 4931–2020 are shown in Figure 6. The result showed that the resultant lignin for all samples had a fixed carbon content above the SNI 4931–2010 standard.

**Figure 7:**
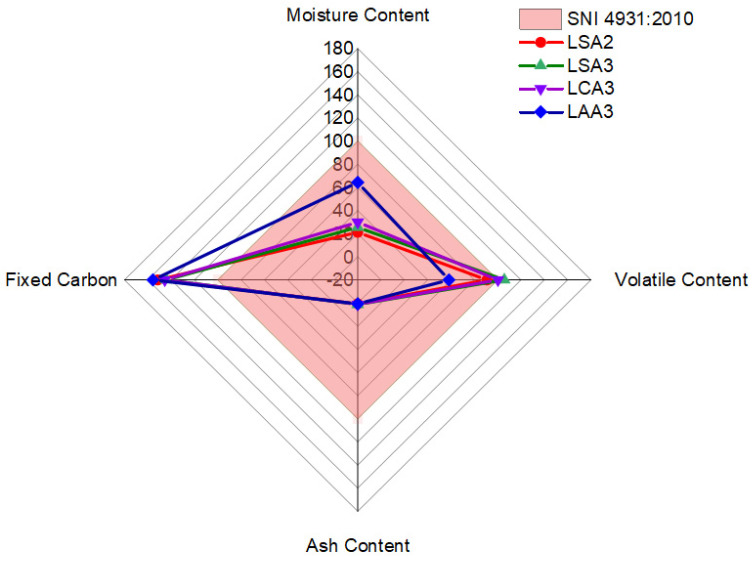
Summary of proximate analysis result.

Summary of proximate analysis evaluation fitted to SNI 4931–2010 standard is presented in Figure 7. The result showed that the LSA2 and LAA3 lignin briquettes samples were in good agreement with the SNI standard 4931–2010, as it had a moisture content, volatile content, and ash content under the maximum allowed value and a fixed carbon content above the minimum requirement.

### 2.3. Effect of Acid Type on Ultimate Analysis of Isolated Lignin

**Figure 8:**
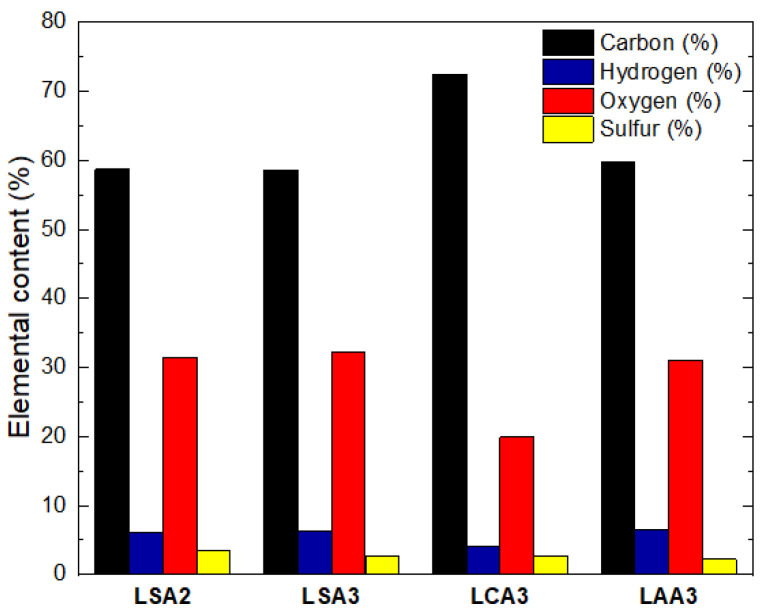
Effect of Acid Type on Ultimate Analysis of LSA2, LSA3, LCA3, and LAA3.

The ultimate test result that consisted of the mass percent of carbon, hydrogen, nitrogen, oxygen, and sulfur in the isolated lignin, is presented in Figure 8. The highest carbon content was obtained from the utilization of citric acid at pH 3 during precipitation, which reached 72.43%.

The percentage of hydrogen and oxygen contained in LCA3 was lower than LSA2, LSA3, and LAA3. This was probably because citric acid had a better ability to dissolve impurities and consisted of various polysaccharide degradation products that had a high amount of hydrogen and oxygen in their structures. To confirm this hypothesis, FTIR characterization was conducted to evaluate functional groups present in the samples.

**Figure 9:**
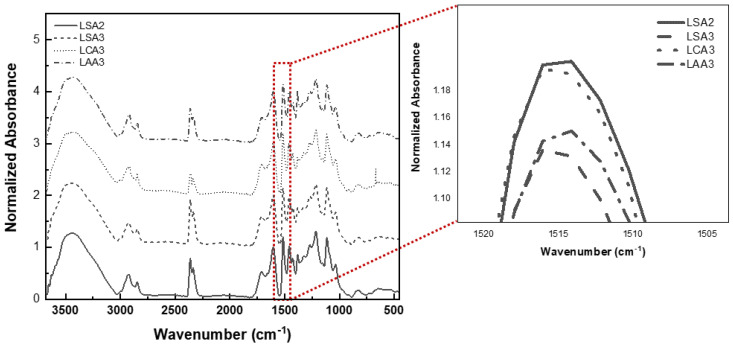
The results of normalized FTIR characterization of LS3, LCA3, and LAA3.

FTIR spectra of the lignin samples are presented in Figure 9. The absorbance pattern of the FTIR spectrum represents the vibrational modes of the samples. The samples showed a broad band centered at 3400 cm^−1^, which attributed to the O-H stretching vibration in lignin. The band around 2900 cm^−1^ and 2850 cm^−1^ indicated the vibration of C-H stretching in the methyl and methylene group, respectively. The band around 1700 cm^−1^ was assigned to the vibration of C=O stretching. The 1600 cm^−1^, 1512 cm^−1^, and 1450 cm^−1^ bands indicated the C=C stretching in aromatic ring. The result showed that LCA3 had a higher number of aromatic groups, as compared to LSA3 and LAA3. On the other hand, the lignin purity of LCA3 was lower than LSA3 but was slightly higher than LAA3. This proved that some of the aromatic rings contained in LCA3 were not aromatic rings that made up lignin. These aromatic groups were probably impurities resulting from the degradation of lignin, such as phenols and several other aromatic derivatives. The C-O stretching of syringil, guacyl, and phenolic groups could be indicated by peaks at 1327 cm^−1^, 1267 cm^−1^, and 1215 cm^−1^, respectively. The absorbance peak in 1100 cm^−1^ and 1035 cm^−1^ indicated the C-H deformation of the aromatic ring and C-O stretching in secondary alcohol.

**Figure 10:**
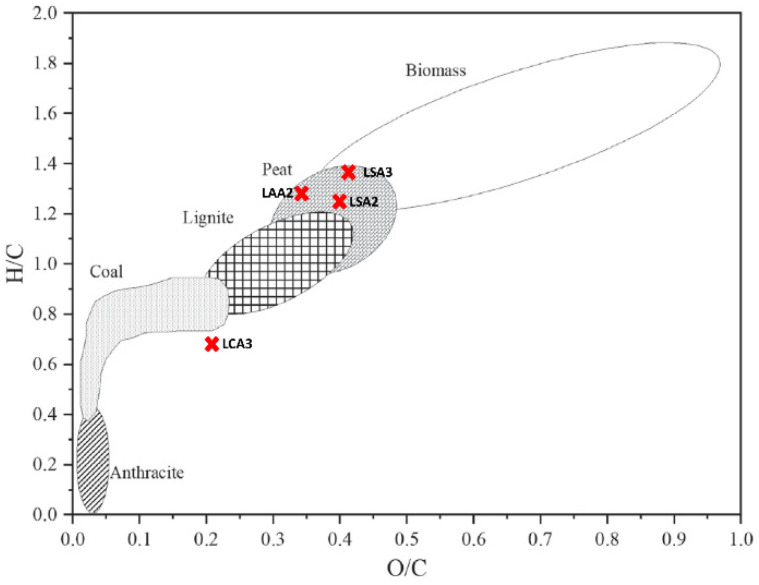
Position of the resultant Briquette on the Van Kravelen diagram (adapted and modified from [31]).

Comparison of the atomic ratio H:C and O:C in the lignin LSA2, LSA3, LCA3, and LAA3 in the Van Kravelen diagram is presented in Figure 10. The result showed that LSA2, LSA3, and LAA3 were included in the peat category, whereas lignin LCA 3 approached the coal category.

### 2.4. Effect of Acid Type on Caloric Value

**Figure 11:**
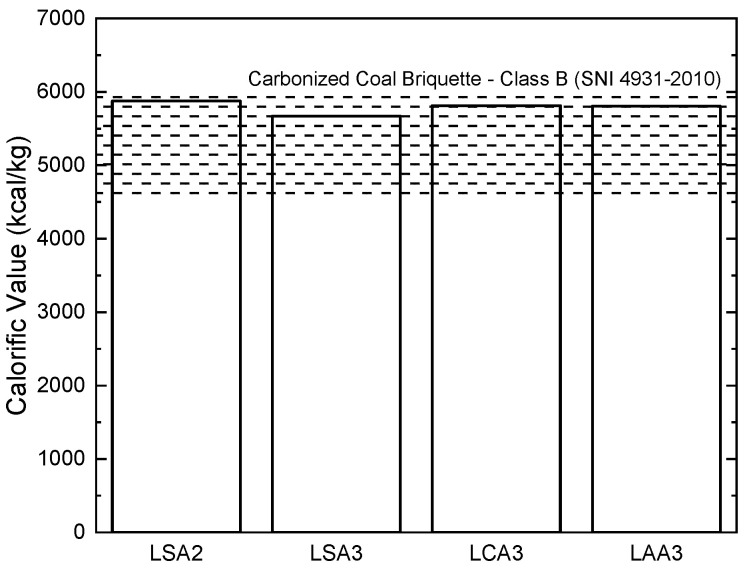
Calorific Value of the Resultant Lignin Briquettes.

The calorific value of the lignin LSA2, LSA3, LCA3, and LAA3 compared to coal briquettes, according to the SNI 4931–2010 standard presented in Figure 11. Based on Figure 11, the highest calorific value resulted in lignin LSA2, which was around 5876 kcal/kg. The overall result showed that all lignin briquette prepared in this research were equivalent to the carbonized coal briquette, according to the SNI 4931–2010 standard.

### 2.5. Effect of Acid Type on Density and the Drop Shatter Index (DSI)

The results of density measurements and the Drop Shatter Index (DSI) of the lignin LSA2, LSA3, LCA3, and LAA3 are shown in Table 2. DSI of all lignin briquettes showed interesting results, with a value above 99%, with DSI of LSI 3 briquette even achieving 100%. All briquettes prepared in this research showed a higher DSI than in commercially available biomass briquette, which was around 96% [32]. This finding showed that lignin could be used to form excellent briquettes even without a binder and was very promising to be utilized as a binderless briquette.

Density measurement showed that the lignin briquette had a density of around 1200 to 1225 kg/m^3^. The highest briquette density was 1225 kg/m^3^ and reached in the LSA3 sample. The density of all obtained briquette was higher than the commercial lignin briquette, which was around 900 to 1000 kg/m^3^ [32]. That condition confirmed its excellent DSI value among the others.

The overall result showed the potential of the resultant binderless, all-lignin briquette to be utilized for energy generation. Lignin briquette also had the potential to be developed into a supercapacitor, if the higher carbon content could be attained, creating another economical value for the resulted lignin briquette.

## 3. Materials and Methods

### 3.1. Materials

The kraft black liquor used in this research was obtained from a pulp and paper industry in Indonesia, which utilized wood from *Acacia manginum*, *Acacia crasicarpa*, and *Pinus sylvetris*. The processed pulp had a kappa number around 16 in the digester and 10, after the oxygen delignification process. Analytical sulfuric acid with 97% purity, citric acid anhydrate, and acetic acid, respectively, was purchased from the SMART LAB, PT. Brataco and *Rofa Laboratorium*, Bandung, Indonesia. Deionized water (DI) used for the lignin purification was purchased from the Chemistry Study Program, ITB, Indonesia. Chemicals used were utilized without further purification.

### 3.2. Method

#### 3.2.1. Isolation of Lignin from Kraft Black Liquor

Lignin from the kraft black liquor was isolated through the acid precipitation process. First, black liquor was dissolved in demineralized (DM) water. Black liquor and water were maintained with a weight ratio of 1:12. Sulfuric acid (H_2_SO_4_) (6M) was added gradually to the diluted black liquor solution, until the pH level was maintained at 2. A similar process was conducted with precipitation pH maintained to 3, 4, and 5. Citric acid anhydrate 50% and acetic acid 99% were also similarly used for precipitation with final precipitation pH levels varying between 3, 4, and 5. The precipitate was filtered from the solution using filter paper and was washed using deionized (DI) water, until a neutral pH was achieved. Lignin precipitates were dried and weighed periodically, until their constant masses were reached. Dried lignin was dispersed with a weight ratio of 1:300 to deionized water in a reflux flask. Finally, lignin was filtered, dried, and weighed periodically, until their constant masses were obtained. Table 3 showed the designation of samples prepared in this research, based on the type of acid used and the pH level was maintained during precipitation.

#### 3.2.2. Briquetting Process

**Figure 12:**
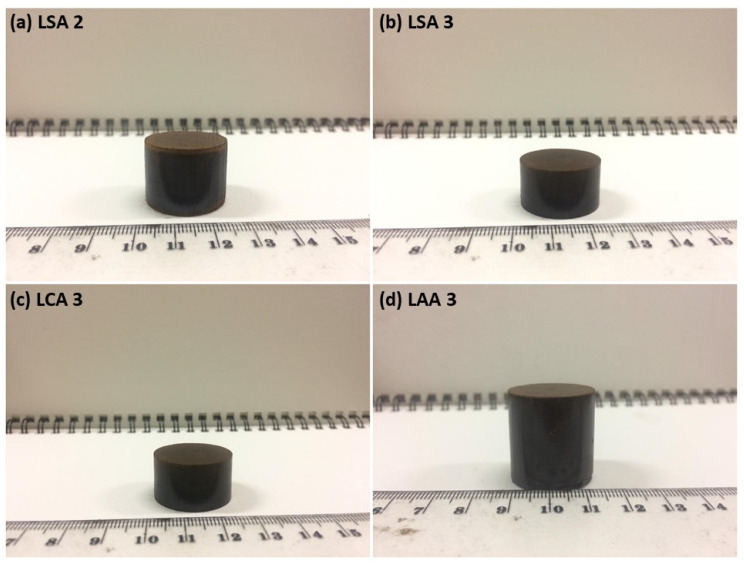
Lignin briquette of (**a**) LSA2, (**b**) LSA3, (**c**) LCA3, and (**d**) LAA3.

The briquetting process was carried out using a hydraulic press and punching tools that consisted of a lower punch, a die, and an upper punch. Briquetting was conducted in the Energy Conversion Lab, Institut Teknologi Bandung, Indonesia. The pressure was maintained to 500 bar, as a result of the calculated minimum pressure required to form a lignin briquette with a certain briquette strength. The briquetting was conducted using a 3.5-tonne hydraulic jack to obtain a 30-mm diameter cylindrical briquette. The briquetting pressure in this process was higher than the conventional briquetting process, with a binder that usually required 100 bar to produce a briquette. A typical binderless briquetting process, such as that used in Komarek, usually requires 500–1000 bar to produce a binderless briquette [33]. The resultant lignin briquettes are shown in Figure 12.

#### 3.2.3. Evaluation of Lignin Yield

The yield of lignin was calculated from the ratio between the resulted dry purified lignin (*b*) to the black liquor used in the process (*a*). Measurement was repeated three times for each variation. The percentage of yield was calculated using Equation (1).

(1) $$\mathrm{Lignin}\;\mathrm{yield}\;=\;\frac{b}{a}\;\times \;100\%$$

#### 3.2.4. Evaluation of Lignin Purity

Evaluation of lignin purity isolated from black liquor was conducted using the Klasson lignin method, according to the TAPPI T-222 OM-02 [34]. Testing was conducted in Metallurgical and Material Engineering Laboratory, Material Engineering Study Program, Institut Teknologi Bandung, Bandung, Indonesia. The testing process used 1 g of lignin (*a*) that dissolved in 15 mL of 72% sulfuric acid and was stirred using a 200-rpm magnetic stirrer for 2 h. The dissolved sample was diluted until the sulfuric acid concentration reached 3% and boiled for 4 h. The precipitate was separated from the solution using the Whatman 42 filter paper. The residue was then washed with DI water to neutral pH, dried, and weighed periodically to a constant weight (*b*). Testing was repeated three times for each variation. The percentage of lignin calculated using Equation (2).

(2) $$\mathrm{Lignin}\;\mathrm{purity}\;=\;\frac{b}{a}\;\times \;100\%$$

#### 3.2.5. Evaluation of Moisture Content

The moisture content of lignin was evaluated according to the ASTM D 3173 [35]. Moisture content was determined by measuring the mass loss of the sample after heating. Lignin sample was weighed (*a*) and placed into a crucible. The sample was dried in an oven heated to 104 °C–110 °C for 1 h. The sample was cooled in a desiccator for 1 h. The dried sample was weighed to determine its final mass (*b*). The sample was measured 3 times for each variation. The percentage of moisture content was determined using Equation (3).

(3) $$\mathrm{Moisture}\;\mathrm{content}\;=\;\frac{\left(a-b\right)}{a}\;\times \;100\%$$

#### 3.2.6. Evaluation of Ash Content

Ash content measurement was carried out according to the ASTM D 3174 [36]. Lignin was weighed to 1 g (*a*) and put into a capsule and then heated in a furnace at 450–500 °C. The temperature was increased to 700–750 °C for 1 h and then increased to 950 °C for 2 h. The residue left after heating was cooled and weighed (*b*). The sample was measured 3 times for each variation. Ash content was calculated using Equation (4).

(4) $$\mathrm{Ash}\;\mathrm{content}\;=\;\frac{b}{a}\;\times \;100\%$$

#### 3.2.7. Evaluation of Volatile Matter

Volatile matter content was evaluated according to the ASTM D 3175 [37]. Lignin was weighed to 1 g (*a*) and placed into a covered platinum crucible. The crucible was put into an inert furnace and heated at 950 °C for 7 min. The sample was cooled in a desiccator after heating. The final mass (*b*) was weighed and the weight loss percentage was determined using Equation (5).

(5) $$\mathrm{Weight}\;\mathrm{loss}\;=\;\frac{\left(a-b\right)}{a}\;\times \;100\%$$

The percentage volatile matter was calculated as the difference between weight loss percent and moisture percent. The sample was tested 3 times for each variation.

#### 3.2.8. Evaluation of Fixed Carbon

Fixed carbon content calculated using Equation (6).

Fixed carbon (%) = 100 − (moisture,% + ash,% + volatile,%)(6)

#### 3.2.9. Evaluation of Gross Calorific Value

The calorific value was determined by burning the sample using an adiabatic bomb calorimeter, according to the ASTM D 5865 [38]. The sample was tested in the Public Services Agency of Mineral and Coal, Bandung, Indonesia.

#### 3.2.10. Drop Shatter Index (DSI) Testing

Drop shatter test was conducted according to ASTM D-440-86 [39]. Initial mass (a) of the briquette was weighed, then the sample was placed into the testing machine and dropped from 1.8 m above a steel plate. The dropped briquette was returned to the machine and the process was repeated one more time. Dropped briquette and its crumbled parts were sieved. Mass of briquette remaining in the sieve counted as the final mass of briquette (b). DSI was calculated using Equation (7).

Drop Shatter Index (DSI), % = (b/a) × 100(7)

#### 3.2.11. Density Measurement

The density of the briquette was calculated from the ratio between mass to volume of the briquette. The volume of the briquette was calculated from the dimension of the briquette, measured using a Vernier caliper. The briquette density was calculated using Equation (8).

(8) $$\mathrm{Density}\;\mathrm{of}\;\mathrm{briquette},\;g/{\mathrm{cm}}^{3}\;=\;\frac{\mathrm{mass}\;\mathrm{of}\;\mathrm{briquette}}{\mathrm{volume}\;\mathrm{of}\;\mathrm{briquette}}$$

#### 3.2.12. Ultimate Testing

Ultimate testing was conducted on the basis of ASTM D3176 [40]. Testing was conducted in the Public Services Agency of Mineral and Coal, Bandung, Indonesia.

#### 3.2.13. FTIR Characterization

The lignin powder was pelletized using KBr and its spectra were recorded in the range of 4000 cm^−1^ to 650 cm^−1^, using Shimadzu Prestige 21. The FTIR measurement was conducted in the Chemistry Study Program, Institut Teknologi Bandung, Indonesia.

## 4. Conclusions

The result of this study demonstrated the capability of lignin isolated from black liquor to be used as binderless, all-lignin briquette. Briquette prepared from lignin isolated using citric acid show promise to be utilized for the application mentioned above, as it had a high fixed-carbon content, excellent DSI, and a decent calorific value that was equivalent to coal-based briquette.

## Figures and Tables

**Table 1:** Proximate and ultimate analysis test results of precipitated lignin.

Proximate Analysis					Ultimate Analysis				
Sample	Moisture Content (%)	Volatile Matter (%)	Ash Content (%)	Fixed Carbon (%)	Carbon (%)	Hydrogen (%)	Nitrogen (%)	Oxygen (%)	Sulfur (%)
LSA2	2.54	19.98	0.11	77.28	58.75	6.13	0.41	31.5	3.48
LSA3	3.06	23.26	0.18	73.5	58.52	6.29	0.36	32.14	2.59
LCA3	3.59	22.05	0.17	74.19	72.43	4.13	0.68	19.90	2.66
LAA3	7.71	12.80	0.10	79.39	59.80	6.44	0.26	31.04	2.18

**Table 2:** DSI and Density measurement of the lignin briquette.

Sample	DSI (%)	Density (kg/m^3^)
LSA2	99.8	1190
LSA3	100	1225
LCA3	99.7	1184
LAA3	99.7	1200

**Table 3:** Sample Designation.

Code	Acid Type	pH
LSA2	Sulfuric acid	2
LSA3		3
LSA4		4
LSA5		5
LCA3	Citric acid	3
LCA4		4
LCA5		5
LAA3	Acetic acid	3
LAA4		4
LAA5		5

## Data Availability

The data presented in this study are available on request from the corresponding author. The data are not publicly available due to institutional restrictions.

## Sample Availability

Samples of the compounds are available from the authors.
